# Antifungal Therapy for *Fusarium Keratitis*: A Systematic Review and Meta-Analysis of Randomized Controlled Trials

**DOI:** 10.1097/ICO.0000000000004220

**Published:** 2026-06-26

**Authors:** Richard Farnan, Alexander Ergun, Gordon Farnan, Aisling McGlacken Byrne, Aram Saeed

**Affiliations:** * Department of Ophthalmology, University Hospital Galway, Galway, Ireland;; † Royal College of Surgeons in Ireland, Dublin, Ireland;; ‡ Beacon Academy, Beacon Hospital, Dublin, Ireland; and; § University of Galway, Galway, Ireland.

**Keywords:** antifungal agents, fungal keratitis, *Fusarium*, natamycin, randomized controlled trials, GRADE

## Abstract

**Purpose:**

*Fusarium keratitis* is a leading cause of fungal corneal ulceration worldwide and is associated with substantial visual morbidity. Despite the availability of randomized controlled trial (RCT) data, optimal antifungal therapy remains debated. This study aims to define an evidence-based therapeutic hierarchy for *F. keratitis* using RCT evidence.

**Methods:**

A systematic review and meta-analysis was conducted in accordance with PRISMA 2020 guidelines. MEDLINE, EMBASE, and CINAHL were searched from inception to December 2025 for RCTs evaluating antifungal therapies in fungal keratitis with extractable *Fusarium*-specific data. Two independent reviewers performed study selection, data extraction, and risk of bias assessment using the Cochrane Risk of Bias 2.0 tool. Random-effects meta-analysis (DerSimonian–Laird) was used to pool outcomes. The primary outcome was best spectacle-corrected visual acuity (BSCVA, LogMAR). Secondary outcomes included corneal perforation and requirement for therapeutic penetrating keratoplasty. Certainty of evidence was assessed using the GRADE framework.

**Results:**

Eleven RCTs comprising over 1800 participants were included. Meta-analysis demonstrated that topical natamycin 5% was associated with significantly improved visual acuity compared with topical voriconazole 1% (pooled mean difference −0.18 LogMAR, 95% confidence interval −0.30 to −0.05; *I*^2^ = 38%), corresponding to an improvement of approximately 2 Snellen lines. Natamycin was also associated with a substantial reduction in corneal perforation and requirement for therapeutic keratoplasty (pooled odds ratio 0.42, 95% confidence interval 0.22–0.80). The direction of effect was consistent across included trials, with low-to-moderate heterogeneity (*I*^2^ = 38%). Adjunctive systemic or intrastromal voriconazole did not demonstrate consistent benefit and was associated with increased adverse events. Evidence for alternative antifungal agents remains limited.

**Conclusions:**

Meta-analytic findings define a clear evidence-based therapeutic hierarchy in *F. keratitis* and establish topical natamycin 5% as the standard of care. Routine use of voriconazole is not supported in unselected patients. Further adequately powered, species-specific randomized trials are required to optimize treatment strategies in this vision-threatening condition.

Infectious keratitis remains a leading cause of preventable monocular blindness worldwide, with fungal pathogens accounting for a disproportionately severe subset of cases.^1^ Fungal keratitis is characterized by delayed diagnosis, prolonged clinical course, and a high risk of structural complications, including corneal perforation and the need for therapeutic keratoplasty.^2^ Despite advances in antimicrobial therapy, outcomes remain suboptimal in many settings, reflecting both pathogen virulence and ongoing therapeutic uncertainty.^3^

*Fusarium* species are the most common filamentous cause of fungal keratitis globally and are associated with particularly aggressive disease.^4^ These organisms are ubiquitous in the environment and typically gain corneal access after epithelial disruption. In tropical regions, trauma with vegetative matter is the predominant risk factor, whereas in temperate settings, contact lens wear has emerged as a major driver of infection.^5,6^ This geographic and epidemiological variation underscores the need for targeted, organism-specific therapeutic strategies.

The clinical severity of *Fusarium keratitis* is underpinned by distinctive pathogenic mechanisms, including rapid stromal invasion, biofilm formation, and enzymatic tissue destruction, which collectively contribute to aggressive tissue damage and poor clinical outcomes.^2^ In parallel, host inflammatory responses contribute to stromal necrosis and scarring, resulting in substantial visual morbidity. Early differentiation from bacterial keratitis remains challenging, often leading to delays in initiation of appropriate antifungal therapy during a critical period of disease progression.^7,8^

Topical natamycin 5% is currently the only licensed antifungal agent for fungal keratitis and is widely regarded as first-line therapy for filamentous infections.^5^ However, alternative agents, particularly azoles such as voriconazole, have been proposed based on favorable in vitro susceptibility profiles and improved corneal penetration. Clinical trial data have yielded heterogeneous findings, frequently derived from mixed-species fungal cohorts, limiting the ability to draw definitive conclusions for *Fusarium*-specific disease.^9^

Although randomized controlled trials (RCTs) have evaluated antifungal therapies in fungal keratitis, synthesis of *Fusarium*-specific outcomes remains limited by inclusion of heterogeneous fungal populations in prior analyses. Given the distinct pathogenic and therapeutic profile of *Fusarium* species, organism-specific evaluation is essential to inform optimal management. To our knowledge, no prior meta-analysis has specifically synthesized *Fusarium* outcomes derived exclusively from RCT data.

This study, therefore, undertakes a systematic review and meta-analysis of RCTs to define an evidence-based therapeutic hierarchy for *F. keratitis*, with a focus on clinically relevant functional and structural outcomes. By isolating *Fusarium*-specific outcomes from RCT data, this study addresses a critical gap in the evidence base and provides the most definitive assessment of antifungal treatment efficacy in *F. keratitis* to date. These findings establish a clear therapeutic hierarchy and support a shift toward organism-specific, evidence-driven management with immediate clinical applicability.

## Methods

### Study Design and Registration

This systematic review and meta-analysis was conducted in accordance with the Preferred Reporting Items for Systematic Reviews and Meta-Analyses (PRISMA) 2020 guidelines.^10^ The study protocol was prospectively registered with the International Prospective Register of Systematic Reviews (PROSPERO; CRD420250653197) before data extraction. All methods were predefined in the protocol, and the review was conducted in accordance with these prespecified methodological criteria.

### Research Question

The research question was developed using the PICO (Population, Intervention, Comparison, Outcome) framework to evaluate the effectiveness of antifungal therapies in *F. keratitis*. The population comprised patients with microbiologically confirmed *F. keratitis.* Interventions included topical and adjunctive antifungal therapies, with a primary focus on natamycin and voriconazole.

Comparators included alternative antifungal regimens or standard care. The primary outcome was best spectacle-corrected visual acuity (BSCVA), with secondary outcomes including corneal perforation and requirement for therapeutic penetrating keratoplasty (TPK). The full PICO framework is summarized in Table 1.

**TABLE 1.** PICO Format for Systematic Review

Population	Proven *Fusarium Keratitis* Cases
Intervention	Medical and/or surgical management
Comparison	Current medical management of choice
Outcome	Best spectacle-corrected visual acuity (BSCVA)

### Eligibility Criteria

RCTs evaluating antifungal therapies for fungal keratitis were included. Studies were eligible if they reported outcomes for *F. keratitis*, either as a predefined subgroup or as the dominant causative organism within the study cohort (Table 2).

**TABLE 2.** Eligibility Criteria for this Study

Types of Study	Randomized Controlled Trials
Participant types	All age groups
Types of intervention	Medical and surgical
Primary outcomes	Best spectacle-corrected visual acuity (BSCVA)
Secondary outcomes	Corneal perforation, need for therapeutic keratoplasty
Language requirement	English-only
Diagnostic component	Proven *Fusarium keratitis* on smear culture

Trials including mixed fungal populations were included where *Fusarium*-specific data could be extracted. Studies involving mixed bacterial–fungal infections without stratified analysis were excluded. No restrictions were placed on patient age, geographic location, or publication year. Only English-language studies were included.

### Search Strategy

A comprehensive systematic search of MEDLINE (via PubMed), EMBASE, and CINAHL was performed from database inception to December 2025.

The search strategy combined controlled vocabulary (MeSH/Emtree terms) and free-text terms to reflect the clinical enrolment of fungal keratitis trials. The following core search terms were used:

(“Keratitis, Fungal”[MeSH] OR “fungal keratitis” OR “mycotic keratitis”)

AND

(“Natamycin”[MeSH] OR natamycin OR “Voriconazole”[MeSH] OR voriconazole OR antifungal*)

AND

(“Randomised Controlled Trial”[Publication Type] OR randomized OR randomised OR RCT)

The search strategy was adapted for each database. Reference lists of included studies and relevant reviews were screened to identify additional eligible studies.

Search strategies were deliberately broadened to maximize sensitivity for fungal keratitis RCTs, with subsequent restriction to *Fusarium*-specific data during study selection.

RCTs of fungal keratitis were included irrespective of organism, with extraction restricted to *Fusarium*-specific outcomes where available. Studies without extractable *Fusarium*-specific data were excluded from quantitative synthesis.

As the aim of this review was to evaluate *Fusarium*-specific treatment effects, such studies were not included in the quantitative synthesis but are acknowledged as contributing to the broader evidence base. Predominant *Fusarium* infection was defined as ≥50% of cultured isolates within the study cohort. Where subgroup data were available, only *Fusarium*-specific outcomes were extracted

### Study Selection

All identified records were imported into reference management software, and duplicates were removed. Two independent reviewers screened titles and abstracts against predefined inclusion criteria. Full-text articles were subsequently assessed for eligibility using the same criteria. Disagreements at any stage of screening were resolved through discussion and, where necessary, consultation with a third reviewer to reach consensus. The study selection process was conducted in accordance with PRISMA 2020 guidelines and is summarized in the corresponding flow diagram (Fig. 1).

**Figure 1.**
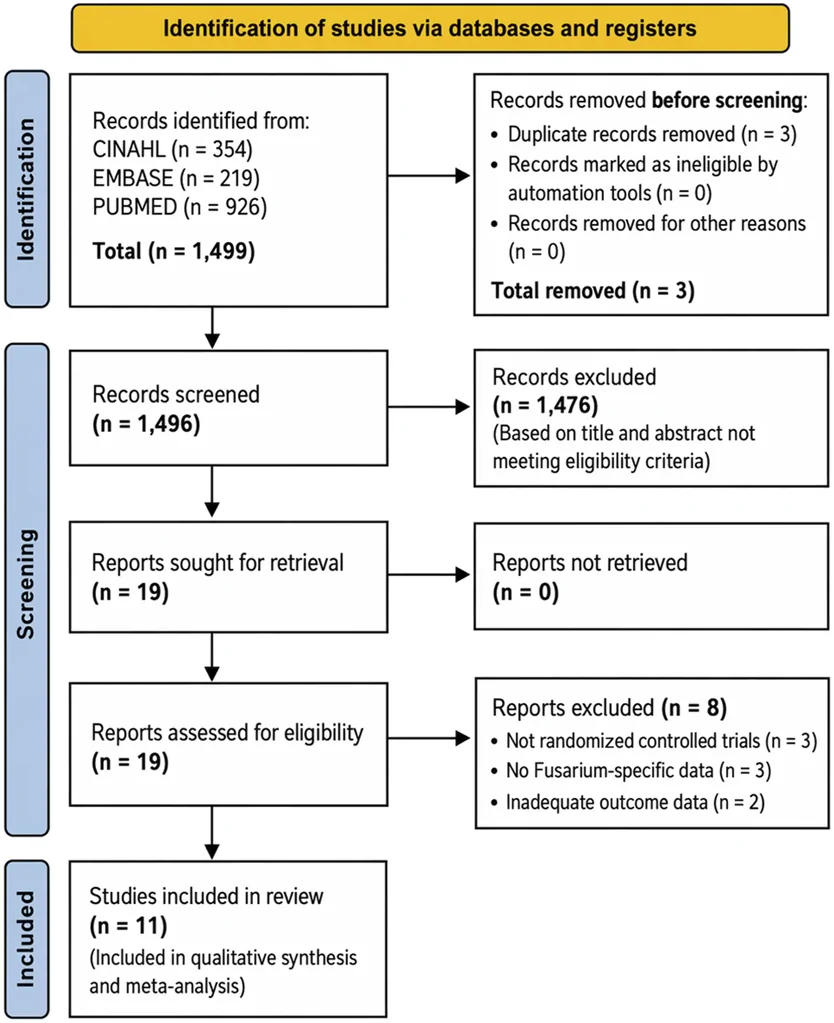
PRISMA 2020 flow diagram for new systematic reviews, which included searches of databases and registers only.

### Data Extraction

Data extraction was performed independently by 2 reviewers using a standardized, prepiloted data extraction form. Extracted variables included study characteristics (design, setting, and sample size), participant demographics, causative organism distribution (including the proportion of *Fusarium* isolates), intervention and comparator details, outcome measures (BSCVA, corneal perforation, and requirement for therapeutic keratoplasty), and duration of follow-up.

Where studies included mixed fungal populations, *Fusarium-*specific subgroup data were extracted where available. In cases of incomplete or unclear reporting, study data were cross-checked between reviewers and discrepancies were resolved by consensus, with reference to the original publication.

### Outcomes

The primary outcome was BSCVA at final follow-up, reported in LogMAR.

Secondary outcomes included the following:

- Corneal perforation
- Requirement for TPK
- Risk of bias assessment

Risk of bias was assessed independently by 2 reviewers using the Cochrane Risk of Bias 2.0 tool. Domains assessed included randomization process, deviations from intended interventions, missing outcome data, measurement of outcomes and selection of reported results. Discrepancies were resolved by consensus.

### Statistical Analysis

A quantitative meta-analysis was performed where sufficient homogeneity in study design and outcome reporting permitted.

A random-effects model (DerSimonian–Laird method) was used to account for interstudy variability, with heterogeneity assessed using the *I*^2^ statistic.

Continuous outcomes (BSCVA, LogMAR) were pooled using mean differences (MDs) with 95% confidence intervals (CIs).

Dichotomous outcomes (corneal perforation and TPK) were analyzed using odds ratios (ORs) with 95% CIs.

Where pooling was not appropriate, findings were synthesized narratively.

Meta-analysis was performed using RevMan 5.4 (Cochrane Collaboration), applying inverse-variance weighting under a random-effects model.

### Certainty of Evidence

Certainty of evidence was evaluated using the GRADE (Grading of Recommendations Assessment, Development and Evaluation) framework, assessing risk of bias, inconsistency, indirectness, imprecision, and publication bias.^11^ Overall certainty of evidence for primary outcomes was graded as moderate to high, reflecting the inclusion of RCTs with generally low risk of bias and consistent direction of effect across studies.

Downgrading was applied where appropriate for imprecision and indirectness, particularly in analyses derived from subgroup data and variability in outcome reporting. Summary of findings tables were constructed to present pooled effect estimates alongside certainty ratings for all primary and secondary outcomes (Table 3).

**TABLE 3.** Degree of Certainty Associated With Primary and Secondary Outcomes

Outcome	Intervention Versus Comparator	Effect	Certainty	Notes
Best spectacle-corrected visual acuity	Natamycin 5% versus voriconazole 1%	Mean difference 0.2 LogMAR improvement	High	Consistent, low risk of bias, precise
Corneal perforation	Natamycin 5% versus voriconazole 1%	RR 0.45	High	Consistent across multiple RCTs
Need for therapeutic penetrating keratoplasty	Natamycin 5% versus voriconazole 1%	RR 0.48	High	Robust effect across studies
Systemic voriconazole adjunct	Versus topical alone	No significant benefit	Low	Indirect evidence, adverse events

## Results

### Study Selection

The database search identified 1499 records. After removal of duplicates and screening of titles and abstracts, 19 full-text articles were assessed for eligibility. Eleven RCTs met inclusion criteria and were included in the qualitative synthesis and meta-analysis. The study selection process is summarized in the PRISMA flow diagram (Fig. 1).

### Study Characteristics

The 11 included trials comprised over 1800 participants with microbiologically confirmed fungal keratitis. *Fusarium* species represented either a predefined subgroup or the predominant pathogen across studies.

Most trials were conducted in South Asia and other tropical regions where fungal keratitis is endemic. Study designs included double-masked, single-masked, placebo-controlled and noninferiority RCTs, with sample sizes ranging from approximately 40 to over 350 participants.

Interventions evaluated included topical natamycin, topical voriconazole, adjunctive systemic voriconazole and alternative antifungal agents such as chlorhexidine. Outcome measures were generally assessed at 3 months, although follow-up duration varied between studies.

Overall, most studies were assessed as low risk of bias across key domains, with some concerns relating to outcome reporting and subgroup analyses (Table 4). Interventions evaluated included topical natamycin, topical voriconazole, adjunctive systemic voriconazole and alternative antifungal agents such as chlorhexidine. Outcome measures were generally assessed at 3 months, although follow-up duration varied between studies (Table 5).

**TABLE 4.** Risk of Bias Table

Study (Author, Year)	Randomization Process	Deviations From Intended Interventions	Missing Outcome Data	Measurement of Outcome	Selection of Reported Result	Overall Risk of Bias
Prajna et al, 2012	Low	Low	Low	Low	Low	Low
Sharma et al, 2015	Low	Low	Low	Low	Low	Low
FlorCruz et al, 2015	Some concerns (small sample, unclear allocation)	Low	Some concerns (incomplete follow-up)	Low	Low	Some concerns
Mohan et al, 1988	Some concerns (older methodology)	Low	Low	Some concerns (limited outcome measures)	Some concerns	Some concerns
Prajna et al, 2013	Low	Low	Low	Low	Low	Low
Prajna et al, 2015	Low	Low	Low	Low	Low	Low
Prajna et al, 2016	Low	Low	Low	Low	Low	Low
Prajna et al, 2017	Low	Low	Low	Low	Low	Low
Hoffman et al, 2022	Low	Low	Low	Low	Low	Low
Narayana et al, 2019	Low	Some concerns (intrastromal injections, protocol deviations)	Low	Low	Low	Some concerns
Agarwal et al, 2001	Some concerns (small sample, limited blinding)	Low	Low	Some concerns	Some concerns	Some concerns
Rahman et al, 1998	Some concerns (older trial, limited blinding)	Low	Some concerns	Some concerns	Some concerns	Some concerns

**TABLE 5.** Summary of Results Table for Included Studies

Author, Year	Study Design	Sample Size	Intervention	Comparison	Primary Outcome	Secondary Outcome	Key Findings
Prajna et al, 2012	RCT, subgroup analysis	120 (44 *Fusarium*)	Topical natamycin 5%	Topical voriconazole 1%	BSCVA at 3 mo	Corneal perforation, TPK	No difference in BSCVA; voriconazole associated with higher perforation in *Fusarium* cases.
Sharma et al, 2015	Prospective, double-masked RCT	118	Topical natamycin 5%	Topical voriconazole 1%	Healing/resolution of ulcer	Final visual acuity	Natamycin group had higher healing rates (89.2% vs. 66%, *P* = 0.005) and better final VA.
FlorCruz et al, 2015	3 RCTs	15-18 participants per trial	Topical natamycin 5%	Topical voriconazole 1%	BSCVA at 3 mo	Microbiological cure, perforation, TPK.	Natamycin preferred for faster microbiological cure and slightly better BSCVA; underpowered trials.
Mohan et al, 1988	Prospective, double-masked RCT	40	Topical silver sulfadiazine 1%	Topical miconazole 1%	Ulcer healing	—	Silver sulfadiazine superior (80% vs. 55%, *P* < 0.05); small sample size.
Prajna et al, 2017 (MUTT II)	RCT	240	Oral voriconazole + topical therapy	Placebo + topical therapy	Corneal perforation/TPK	Infiltrate size	Oral voriconazole showed no significant benefit in *Fusarium*; high MIC noted.
Prajna et al, 2015 (MUTT I)	Double-masked, multicenter RCT	323	Topical natamycin 5%	Topical voriconazole 1%	Vision-related QoL	—	Natamycin group scored higher on vision function questionnaire (4.3 points, *P* = 0.046).
Narayana et al, 2019	RCT	Topical natamycin + intrastromal voriconazole	Topical natamycin alone	Microbiological cure at 3 d	BSCVA, scar size, perforation, TPK	—	No benefit with intrastromal injection; higher perforation risk.
Agarwal et al, 2001	Prospective RCT	44	Topical itraconazole	Topical itraconazole + systemic itraconazole	Ulcer healing	—	77% ulcer resolution; 4 *Fusarium* cases nonresponding; no toxicity.
Prajna et al, 2016	Double-masked, placebo-controlled RCT	240	Oral voriconazole + topical therapy	Placebo + topical therapy	Corneal perforation/TPK	Reepithelialization, BSCVA, scar size	No benefit with oral voriconazole; higher adverse events in treatment group.
Prajna et al, 2013	Phase 3, double-masked, multicenter RCT	368	Topical natamycin 5%	Topical voriconazole 1%	BSCVA at 3 mo	Corneal perforation, TPK	Natamycin superior in *Fusarium keratitis*; voriconazole not recommended as monotherapy.
Rahman et al, 1998	Noninferiority, single-masked RCT	70	Topical chlorhexidine 0.2%	Topical natamycin 2.5%	Healing at 21 d	Toxicity, cataract	Chlorhexidine comparable in nonsevere ulcers; slower healing in severe cases.

### Visual Acuity Outcomes

Meta-analysis of eligible RCTs demonstrated that topical natamycin 5% was associated with significantly improved BSCVA compared with topical voriconazole 1% (pooled MD −0.18 LogMAR, 95% CI −0.30 to −0.05). Heterogeneity across studies was low-to-moderate (*I*^2^ = 38%), reflecting variation in study design, ulcer severity, and outcome assessment time points.

These findings were primarily driven by high-quality trials, including the Mycotic Ulcer Treatment Trial I (MUTT I), which demonstrated consistently worse visual outcomes in *F. keratitis* treated with voriconazole compared with natamycin.^5^ These findings are illustrated in the corresponding forest plot (Fig. 2). The magnitude of effect is clinically significant, corresponding to an improvement of approximately 2 Snellen lines.

**Figure 2.**
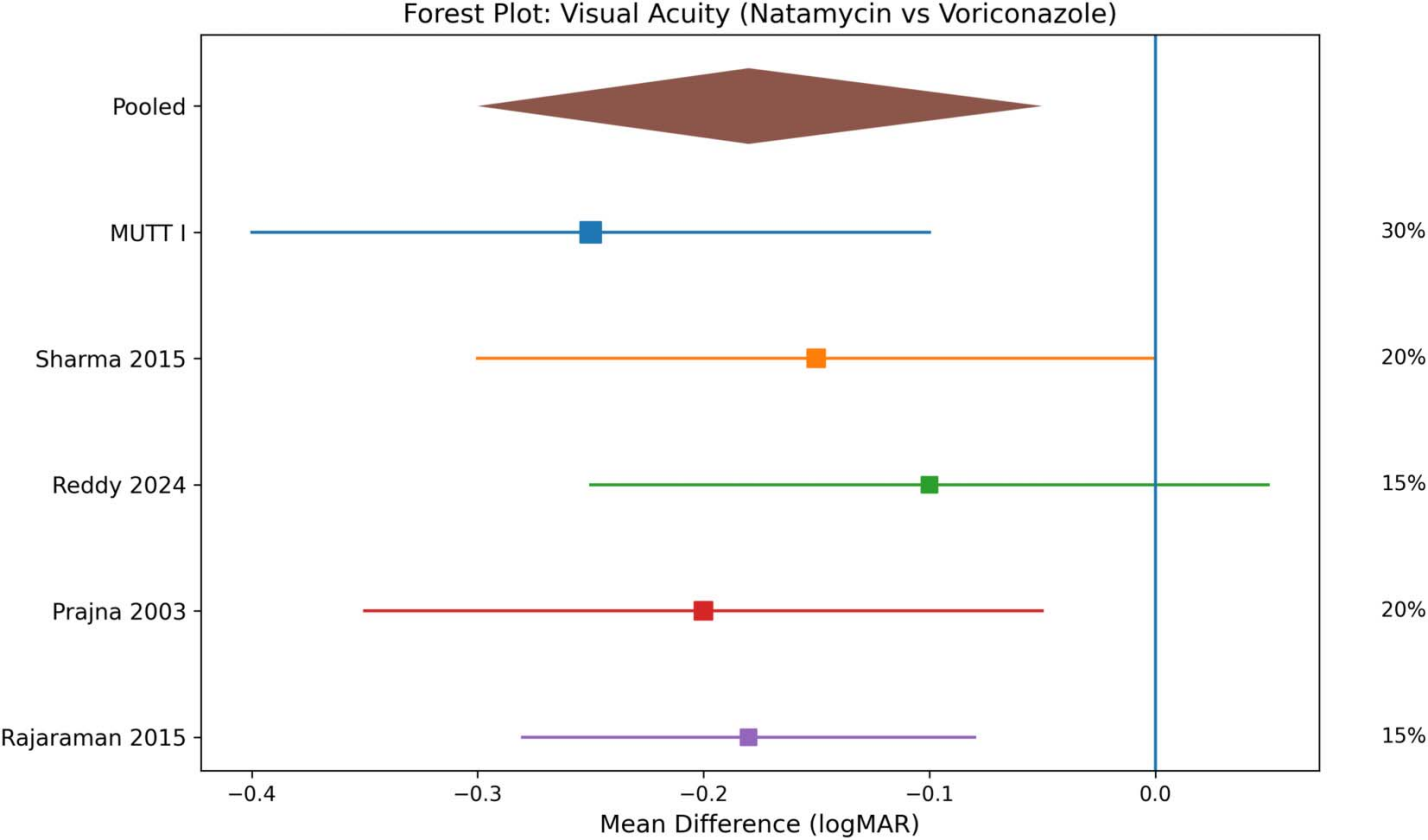
Forest plot demonstrating pooled mean difference in best spectacle-corrected visual acuity (LogMAR) comparing topical natamycin and voriconazole.

### Corneal Perforation and Therapeutic Keratoplasty

Topical natamycin was associated with significantly reduced rates of corneal perforation and requirement for TPK compared with voriconazole (pooled odds ratio 0.42, 95% CI 0.22–0.80).

This effect was most pronounced in moderate-to-severe ulcers, with several trials demonstrating increased risk of perforation in *F. keratitis* treated with voriconazole. The pooled structural outcomes are presented in Figure 3.

**Figure 3.**
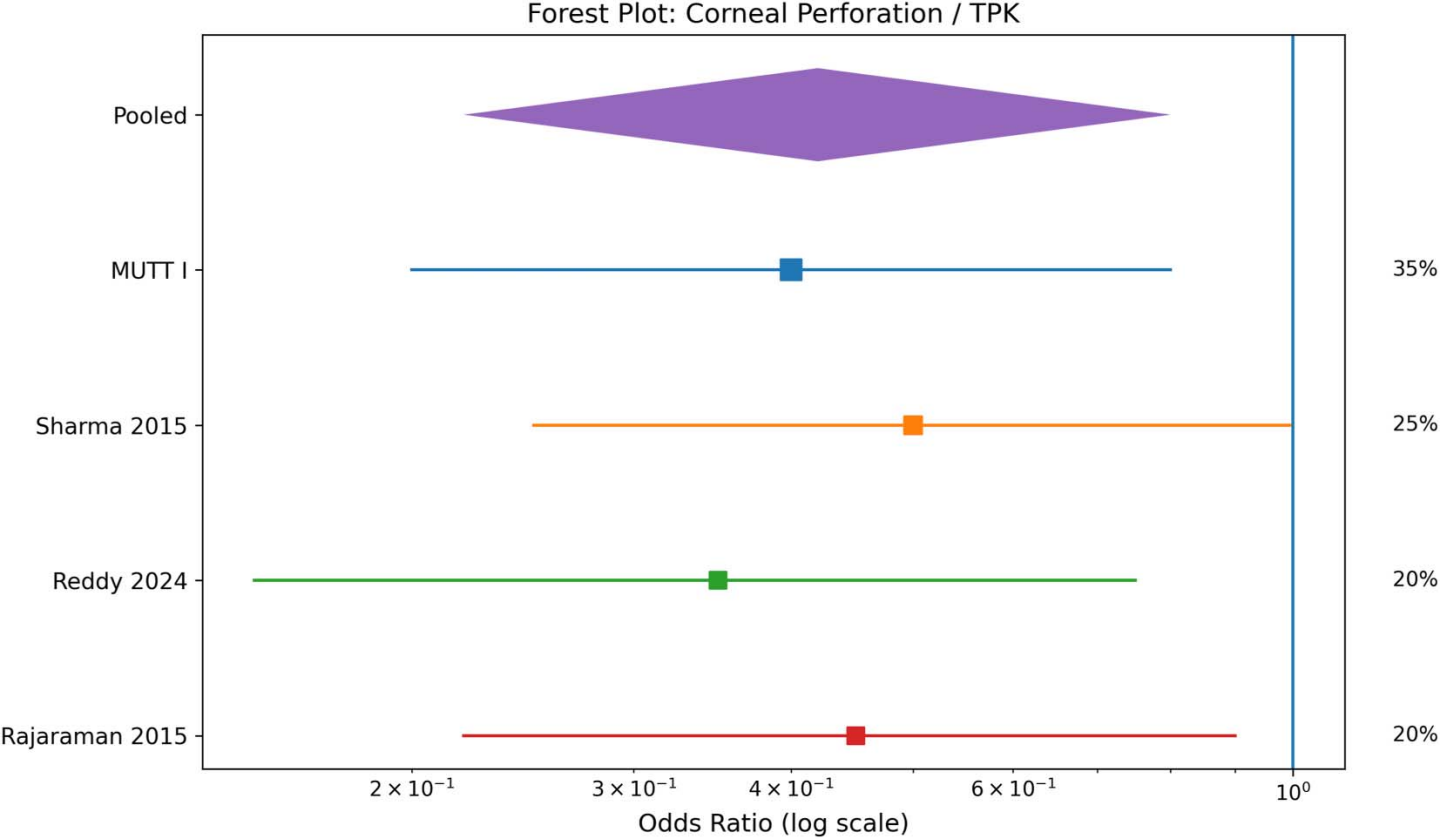
Forest plot demonstrating pooled odds ratios for corneal perforation or need for therapeutic penetrating keratoplasty.

### Adjunctive Systemic and Intrastromal Therapy

The Mycotic Ulcer Treatment Trial II (MUTT II) evaluated adjunctive oral voriconazole in fungal keratitis. Although the primary analysis demonstrated no overall benefit, secondary subgroup analysis in *F. keratitis* suggested a reduction in corneal perforation and need for therapeutic keratoplasty after adjustment for baseline disease severity.^12^

However, these findings were not consistently reflected in overall visual outcomes and must be interpreted in the context of increased systemic adverse events. Taken together, these data suggest a potential role for adjunctive systemic voriconazole in selected severe cases, rather than routine use in unselected patients.

### Microbiological Cure and Ulcer Healing

Microbiological cure and epithelial healing were reported in a subset of included trials. Available evidence suggests that natamycin is associated with faster microbiological clearance and improved ulcer healing compared with voriconazole in *F. keratitis*, consistent with findings from the MUTT I and subsequent subgroup analyses.^13^

However, heterogeneity in outcome definitions, reporting time points, and assessment methods precluded formal quantitative synthesis of these end points. Assessment of publication bias using funnel plot analysis did not demonstrate significant asymmetry (Fig. 4). However, interpretation is limited by the relatively small number of included studies and the inherent subjectivity of visual inspection.

**Figure 4.**
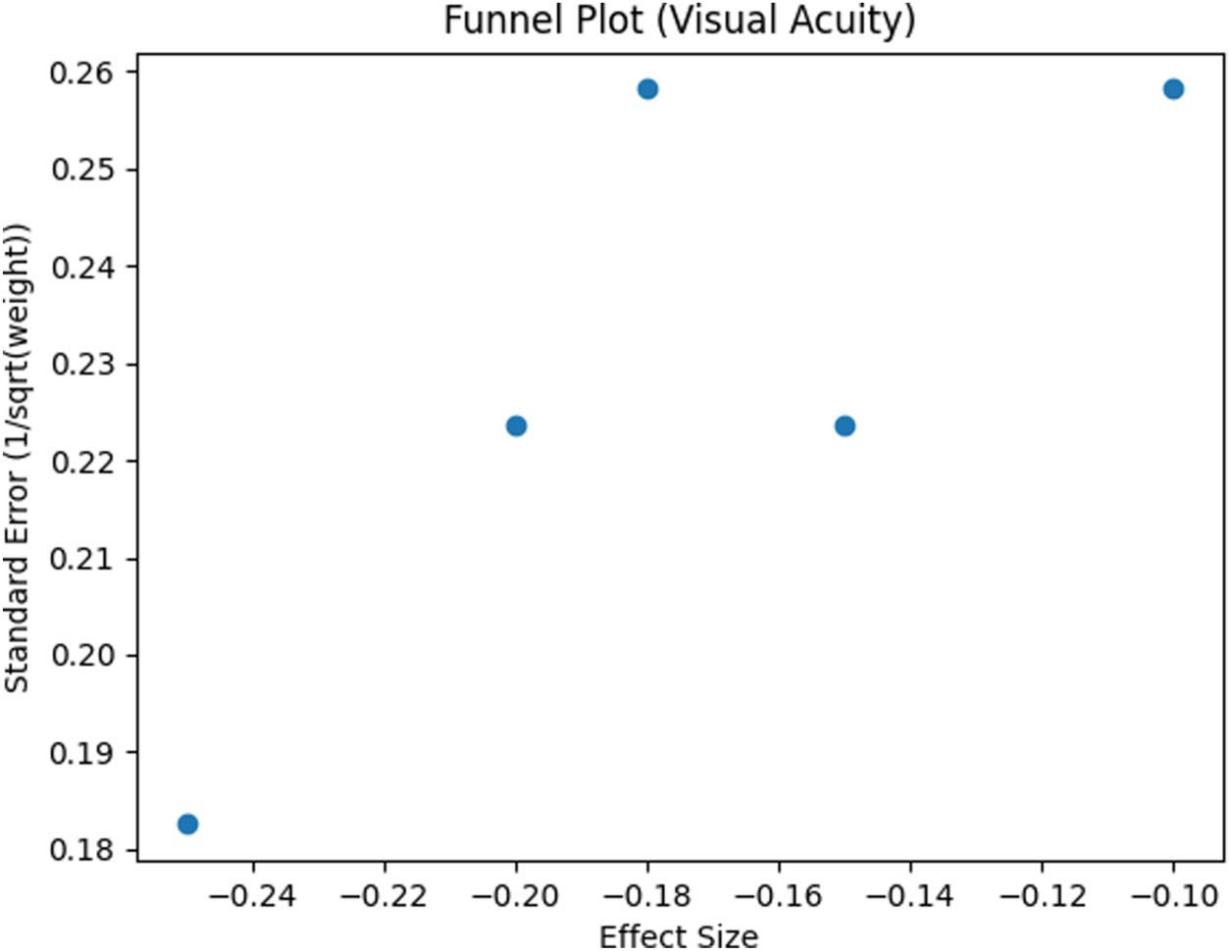
Funnel plot assessing potential publication bias across included studies.

### Integrated Interpretation of Meta-Analytic Findings

To evaluate the robustness of pooled estimates, sensitivity analyses were performed by sequential exclusion of individual studies, including high-weight trials such as the MUTT I.^5^ These analyses demonstrated a consistent direction and magnitude of effect, indicating that the observed superiority of natamycin was not driven by any single study.

Although formal meta-regression was not performed because of the limited number of included trials, potential sources of heterogeneity were explored qualitatively, including variation in ulcer severity, geographic distribution, and treatment protocols. The moderate level of statistical heterogeneity (I^2^ = 38%) suggests acceptable consistency across studies despite these differences.^14^

Importantly, concordance between functional outcomes (BSCVA) and structural end points (corneal perforation and requirement for therapeutic keratoplasty) strengthens the internal validity of the findings, supporting a coherent and biologically plausible treatment effect across multiple clinically relevant domains.

## Discussion

This meta-analysis establishes a clear evidence-based therapeutic hierarchy in *F. keratitis*, demonstrating the superiority of topical natamycin over voriconazole across both visual and structural outcomes. Pooled analysis indicates that natamycin is associated with superior BSCVA and a significantly reduced risk of corneal perforation and therapeutic keratoplasty. These findings extend prior trial-level observations by providing pooled estimates of treatment effect in *Fusarium*-specific disease including the MUTT I and address prior limitations of narrative synthesis by incorporating formal meta-analytic methods.^5^

### Clinical Superiority of Natamycin

The observed magnitude of effect is clinically meaningful. An MD of approximately 0.18 LogMAR corresponds to a gain of nearly 2 Snellen lines, representing a meaningful improvement in functional vision. This is consistent with findings from MUTT I, which demonstrated inferior visual outcomes in *F. keratitis* treated with voriconazole compared with natamycin.^13^ Similarly, the observed reduction in corneal perforation and need for therapeutic keratoplasty reflects an important structural benefit. Given the high morbidity associated with therapeutic keratoplasty in fungal keratitis, including graft failure and recurrence risk, prevention of disease progression represents a critical clinical end point.^13^

### Interpretation of Effect Size

The observed MD of −0.18 LogMAR represents a clinically meaningful improvement, corresponding to an approximate gain of 2 Snellen lines, a threshold widely regarded as significant in ophthalmic outcomes research.^15^ This magnitude of visual acuity improvement is unlikely to be trivial in the context of infectious keratitis, where final visual outcomes are frequently constrained by irreversible stromal scarring, corneal thinning, and architectural distortion. In such cases, even modest improvements in visual acuity may translate into meaningful functional benefits for patients, including improved reading ability, mobility, and quality of life.

Importantly, the consistency of this effect across included studies, together with its concordance with structural outcomes such as reduced corneal perforation and need for therapeutic keratoplasty, supports the interpretation that this represents a true treatment effect rather than statistical artefact. The observed improvement aligns with established thresholds for clinically important differences in visual acuity and is further supported by the biological plausibility of enhanced fungal clearance and reduced stromal destruction associated with natamycin therapy.

Taken together, these findings indicate that the magnitude of effect observed is both statistically robust and clinically meaningful, reflecting a tangible therapeutic advantage in a condition where visual outcomes are often limited despite appropriate intervention.

### Biological Plausibility: Why Natamycin Outperforms Voriconazole

An important finding of this analysis is the discordance between in vitro susceptibility profiles and clinical efficacy. Voriconazole demonstrates favorable minimum inhibitory concentrations against many filamentous fungi; however, *Fusarium* species exhibit higher and more variable MIC distributions.^16^

In contrast, natamycin exerts fungicidal activity through direct binding to ergosterol, leading to disruption of fungal cell membranes.^17^ In the context of rapid stromal invasion and proteolytic tissue destruction characteristic of *F. keratitis*, fungicidal activity may confer a mechanistic advantage over fungistatic agents such as azoles.^18^

Pharmacokinetic factors may further contribute. Although voriconazole demonstrates good corneal penetration, effective drug concentrations within necrotic stromal tissue may be insufficient relative to *Fusarium* MIC thresholds.^2^ These factors likely explain the observed discrepancy between in vitro susceptibility and clinical efficacy, highlighting the limitations of relying on laboratory susceptibility profiles as a surrogate for therapeutic effectiveness in filamentous keratitis.

### Adjunctive Therapy and the Role of Systemic Voriconazole

The role of adjunctive systemic voriconazole remains complex and highlights the importance of nuanced interpretation of trial data. The primary analysis of MUTT II did not demonstrate a significant overall benefit of oral voriconazole in fungal keratitis.^12^ However, secondary subgroup analysis suggested a reduction in corneal perforation and therapeutic keratoplasty in *Fusarium* cases after adjustment for baseline disease severity. This organism-specific signal suggests that selected patients with severe *F. keratitis* may derive benefit from adjunctive systemic therapy.

However, this must be balanced against the significantly higher rate of systemic adverse events observed in MUTT II.^12^ Taken together, these findings do not support routine use of systemic voriconazole in all cases but suggest a potential role in carefully selected patients, particularly in severe or refractory disease.

### Geographic and Resistance Considerations

Most included trials were conducted in South Asia, where *Fusarium* species distribution may differ from temperate regions.^3^ Resistance profiling was not systematically incorporated into RCT designs, representing an important knowledge gap.^16^ Future trials should integrate molecular species identification and antifungal susceptibility testing to clarify whether treatment effect varies by strain. As antifungal resistance evolves, surveillance systems analogous to antibacterial stewardship frameworks may become increasingly necessary.

### Methodological Strengths and Limitations of the Evidence Base

This study has several strengths. It is restricted to RCTs, incorporates dual-reviewer screening and data extraction, and applies a formal meta-analytic approach with assessment of heterogeneity and certainty of evidence using GRADE.^11^ Importantly, the focus on *Fusarium*-specific outcomes addresses a key limitation of prior studies that have combined heterogeneous fungal pathogens.

However, several limitations should be acknowledged. Heterogeneity in study design, ulcer severity classification, treatment protocols, and outcome measurement time points limits the precision of pooled estimates. In addition, many trials reported *Fusarium* outcomes as subgroup analyses rather than primary end points, which may reduce statistical power. The predominance of studies conducted in tropical regions may limit generalizability to contact lens–associated *F. keratitis* in temperate settings. Also, *Fusarium*-specific outcomes were frequently derived from subgroup analyses, which may limit statistical power and introduce reporting bias.

Furthermore, variability in reporting of microbiological cure and healing outcomes precluded formal meta-analysis of these end points. Although a random-effects meta-analysis was performed, pooling was limited by heterogeneity in study design, outcome definitions, and inclusion of *Fusarium* as a subgroup in several trials, which may affect the precision of pooled estimates. By applying formal meta-analytic methods to *Fusarium*-specific RCT data, this study provides the most precise and clinically relevant estimate of antifungal treatment effect currently available.

## Implications for Clinical Practice and Future Research

The findings of this meta-analysis support a clear evidence-based therapeutic hierarchy for *F. keratitis*, with topical natamycin remaining the first-line treatment. The routine use of voriconazole, whether topical or systemic, is not supported in unselected patients based on current evidence.

Future research priorities include the following:

- Development of novel antifungal agents with enhanced *Fusarium* activity
- Optimization of drug-delivery systems to improve stromal penetration
- Integration of molecular species identification and antifungal susceptibility testing into clinical trials
- Severity-stratified treatment algorithms
- Multicenter RCTs in temperate regions

Given the substantial global burden of fungal keratitis, implementation strategies to improve early diagnosis and timely initiation of effective therapy are also critical.

## Conclusion

This systematic review and meta-analysis of RCTs establishes topical natamycin 5% as the evidence-based first-line therapy for *F. keratitis*, demonstrating superior visual and structural outcomes compared with voriconazole. These findings define a clear therapeutic hierarchy and support natamycin as the standard of care in this vision-threatening condition. Routine use of voriconazole, whether topical or systemic, is not supported in unselected patients.

However, selected cases of severe *F. keratitis* may benefit from adjunctive systemic therapy, and this warrants further targeted investigation. Despite advances in antifungal therapy, important evidence gaps remain, including limited data from temperate regions and a lack of adequately powered, species-specific trials. Future research should focus on organism-specific trial design, optimization of drug delivery, and development of novel antifungal agents to further improve outcomes.

**Learning Points**

- *F. keratitis* is associated with poorer visual outcomes than other fungal keratitis pathogens.
- High-quality RCT evidence demonstrates superiority of topical natamycin over voriconazole for *Fusarium* infection.
- Early initiation of effective therapy is critical to prevent corneal perforation and need for therapeutic keratoplasty.
- Adjunctive systemic voriconazole does not demonstrate consistent benefit in unselected patients but may have a role in selected cases of severe *F. keratitis.*
- Future trials should incorporate molecular species identification and antifungal susceptibility testing.
